# Fixation duration surpasses pupil size as a measure of memory load in free viewing

**DOI:** 10.3389/fnhum.2014.01063

**Published:** 2015-01-21

**Authors:** Radha Nila Meghanathan, Cees van Leeuwen, Andrey R. Nikolaev

**Affiliations:** Laboratory for Perceptual Dynamics, Faculty of Psychology and Educational Sciences, University of LeuvenLeuven, Belgium

**Keywords:** eye movements, fixation duration, pupil size, memory load, processing load, attention, working memory, visual search

## Abstract

Oculomotor behavior reveals, not only the acquisition of visual information at fixation, but also the accumulation of information in memory across subsequent fixations. Two candidate measures were considered as indicators of such *dynamic* visual memory load: fixation duration and pupil size. While recording these measures, we displayed an arrangement of 3, 4 or 5 targets among distractors. Both occurred in various orientations. Participants searched for targets and reported whether in a subsequent display one of them had changed orientation. We determined to what extent fixation duration and pupil size indicate dynamic memory load, as a function of the number of targets fixated during the search. We found that fixation duration reflects the number of targets, both when this number is within and above the limit of working memory capacity. Pupil size reflects the number of targets only when it exceeds the capacity limit. Moreover, the duration of fixations on successive targets but not on distractors increases whereas pupil size does not. The increase in fixation duration with number of targets both within and above working memory capacity suggests that in free viewing fixation duration is sensitive to actual memory load as well as to processing load, whereas pupil size is indicative of processing load only. Two alternative models relating visual attention and working memory are considered relevant to these results. We discuss the results as supportive of a model which involves a temporary buffer in the interaction of attention and working memory.

## Introduction

Vision science is shifting its focus from the traditional stimulus-response paradigm to a more ecologically valid approach: the analysis of continuous visual processes in free viewing. In these conditions, visual information is acquired via eye movements and thus their measurement may provide crucial insights into the time course of processing and accumulation of visual information.

In freely viewing, the eyes are directed to different locations of interest via saccades. The eyes fixate these locations in order to identify and encode information (Henderson and Hollingworth, 1999; Irwin, 2004). Over time, information from the fixated locations is accumulated in memory across multiple fixations (Melcher, 2001; Hollingworth and Henderson, 2002; Tatler et al., 2003). In order to study accumulation of information in memory in free viewing conditions, it is important to determine which measures of eye movement are sensitive to memory load. Such measures could be useful in combination with behavioral responses, but also in combination with neurophysiological recording, for example, of electrical brain activity.

One of the most common and informative eye movement measures is fixation duration. The duration of fixation increases under processing load, i.e., it increases when processing becomes more effortful (Inamdar and Pomplun, 2003; Peterson et al., 2008; He and McCarley, 2010). In particular, fixation duration is sensitive to the amount of attention deployed to a fixated location (Just and Carpenter, 1980; Irwin, 2004; Henderson, 2007) and, crucially, memory load lengthens the average duration of fixation in free viewing conditions, as shown in visual search studies (McCarley et al., 2006).

Another potentially useful information processing indicator is pupil size. When measured at fixation, the pupillary response increases with memory accumulation and task difficulty (Beatty, 1982). Pupil size saturates at the limit of working memory capacity and decreases during use of memory strategies that reduce load (Beatty, 1982; Andreassi, 2000; Beatty and Lucero-Wagoner, 2000). Pupil size is relatively unexplored, however, as a measure in free viewing conditions. We are aware of only one study where pupil size increased with memory load during unrestricted eye movements (Porter et al., 2007). In their study, the pupil dilated more for larger search set size, larger number of search targets, and for heterogeneous as compared to homogeneous distractors.

This study aims at assessing fixation duration and pupil size, specifically, as measures of *accumulating* memory load in free viewing conditions. Whereas previous studies considered the effect of memory load on eye movement measures averaged across the duration of free viewing (McCarley et al., 2006), here we will focus on how these measures change dynamically as memory load increases as participants gather information during viewing of a display. To do this, we used a visual search task with multiple targets. The presence of multiple targets necessitated them to be accumulated in memory during free viewing. The visual search was followed by a change detection task in order to assess memory for the targets. The number of targets in a display was varied between 3, 4 and 5, a number chosen to lie around the working memory capacity limit of 4 (Luck and Vogel, 2013). We predict that both fixation duration and pupil size will reflect the number of targets in the search task. Moreover, these measures might also reflect the number of distractors viewed, to the extent that they enter memory.

Besides memory load, the task also involves attentional selection, control operations, and other cognitive processes. Fixation duration and pupil size may, in principle, be differently sensitive to all of these processes and their interactions. To predict the interaction of these processes and memory load during the task, we draw on two recent models.

According to Model I (Bowman and Wyble, 2007; Wyble et al., 2011, 2015), limited resources are shared between working memory and attention. This implies that during the course of a search task, as each new target is detected, working memory becomes increasingly loaded, resulting in decreasing processing rate. Therefore, in our visual search task, a difference between 3, 4 and 5 targets should be seen from early on. According to Model II (Simione et al., 2012; Raffone et al., 2014), new targets will be loaded automatically into a temporary global workspace buffer with limited capacity. When this buffer is full, control operations involving interactions between attention, working memory and global workspace are needed, in order to select and manage the content for consolidation in memory. These interactions give rise to additional processing load. Therefore, in the early stage of a search task, effects of memory load alone will be obtained. When the buffer is loaded to capacity, additional effects of attentional and processing load will appear. Hence according to Model II, a difference between 3 and 4 targets should be seen early, since they lie within memory capacity limit. But, in addition, a difference between 4 and 5 targets would arise with a late onset, because of the additional processing load that becomes necessary when the buffer is full. This prediction contrasts with Model I. Both models have in place an early mechanism of attentional filtering, which warrants that only targets are loaded into working memory. Therefore, the effect of memory load will be seen only in target processing and not on distractor processing.

To evaluate fixation duration and pupil size as memory load measures, we test the extent to which the two measures adhere to one of these alternative models. The degree of conformity with one of the models would legitimate the measure; at the same time it would support the model to which it adheres.

## Material and methods

### Participants

Twenty-three participants (ages 18–29 years, mean 20.86 years, 7 male) took part in the experiment. Of these, 15 reported normal vision, 6 had their vision corrected to normal with glasses and two with contact lenses. Two participants were excluded because of noisy eye-movement data and two others for failure to meet the criterion number of trials in each condition as explained below, leaving 19 participants (6 male), whose data were used for the final analysis. All participants gave their written consent. The study was approved by the departmental Ethics Committee of the KU Leuven.

### Stimuli and apparatus

**Figure 1:**
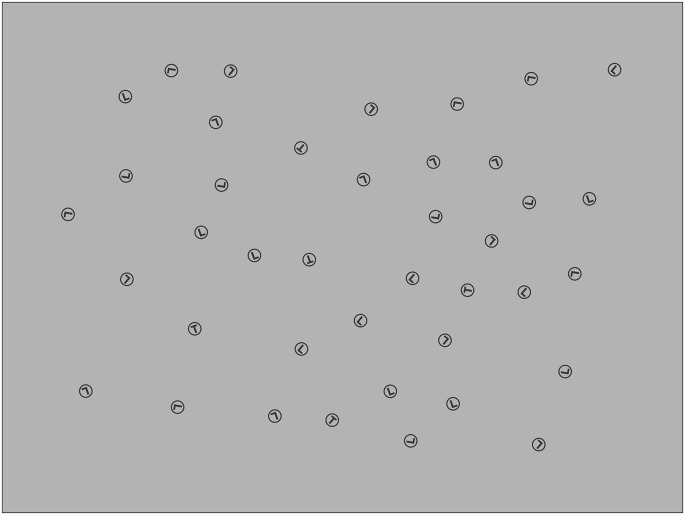
**A sample display with 40 items: 5 targets (“T”) and 35 distractors (“L”)**.

We used displays with target and distractor items in various orientations (Figure 1). There were 40 items in each display, 3, 4 or 5 of which were targets. In a pilot experiment using a staircase procedure, we found 40 items to be the optimal set size for 70% correct responses in our task. To avoid any luminance differences between conditions, we kept the number of items constant at 40 across all conditions, while varying the number of distractors as 40 minus the number of targets in the display.

The target stimuli were “T”s, 0.41° × 0.41° of visual angle, and the distractor stimuli were “L”s, 0.31° × 0.41° of visual angle. Both types of items were rotated 20°, 80°, 140°, 200°, 260° or 320°. No item was in vertical or horizontal orientation and no two targets in a display had the same orientation. Each item was surrounded by a circle subtending 0.83° of visual angle. This was done in order to make it too difficult for our participants to discriminate targets without fixating on them (Peterson et al., 2001; Körner and Gilchrist, 2007).

The stimuli were presented in black (0.48 cd/m^2^) on a gray background (32.84 cd/m^2^). The gray background spanned the entire monitor screen and subtended 39.9° × 30.5° of visual angle. The stimuli themselves appeared within a virtual rectangle of size 32.9° × 23.12°. Distractor locations were chosen randomly within this rectangle under the constraint that the minimum distance between two items was 3.12°. Target locations were chosen randomly but with the constraint that targets appeared within a donut shaped region of inner radius 3.12° and outer radius 10.4° from the center of the display, with a probability of 0.86, 0.9 and 0.92 in the 3-, 4- and 5-target conditions, respectively. This was done in order to deter easy detection of targets close to the central fixation dot or near the border of the display by reducing the odds of targets occurring there. Two targets were always separated by a minimum of 6.24° so as to prevent two targets from being detected in a single fixation. Stimuli were presented on a 40 cm × 30 cm monitor with a refresh rate of 75 Hz and a screen resolution of 1600 × 1200 pixels. The viewing distance was 55 cm. The stimulus presentation program was written in Python 2.7.

### Procedure

We used a change-detection task, in which participants searched a first display, the *search display*, for 10 s. This display was followed by a *change detection* display where, in half of the trials, one of the targets (*changed* target) had changed orientation (*different* trials), and in the other half, a display identical to the search display was shown (*same* trials). Participants were asked to report whether a change had occurred.

Each trial comprised of a blank screen with a fixation dot, a *search display*, another blank screen with a fixation cross, a *change detection display*, and a *feedback display*. At the start of each trial, participants were asked to fixate on the central dot and press the space bar to start the task. Immediately after the space bar was pressed, a visual search display was presented for 10 s. Participants were not informed about the number of targets in the display, but had been told in advance that each display would contain 3–5 targets. Participants were asked to search for the targets and memorized the orientation of each target in order to perform change detection in the subsequent display. After the first display, the fixation cross was shown for a duration randomly varied according to a uniform distribution in the interval of 1–1.5 s. The *change detection display* was shown afterwards until participants responded or after another 10 s, whichever was earlier. Participants were asked to respond with the left arrow key to indicate “change” or the right arrow key to indicate “no change”. The response keys were counterbalanced across participants. After response, a feedback screen was displayed for 0.8 s with the targets encircled in green for correct responses and in red for incorrect ones. The feedback for a changed target was given by substituting the response circle around the target with a larger one. The new trial started immediately after the feedback screen.

Participants performed a practice block of 12 trials before the experiment. They were then asked to complete ten blocks of 27 experimental trials each for a total of 270 trials, lasting around 100 min.

### Eye movement and pupil size recording

The desktop system of the EyeLink 1000 eye tracker (SR Research Ltd.) was used for recording eye movements and pupil size. The accuracy of the system is typically between 0.25° and 0.5° and pupil size resolution is 0.2% of diameter. The system records pupil area or diameter as integer numbers in arbitrary units using centroid or ellipse fitting for pupil detection. In our experiment, pupil area was recorded and ellipse fitting was used in order to reduce dependance of pupil size on gaze direction. Eye position and pupil size were tracked at a sampling frequency of 250 Hz.

Participant’s head was stabilized using a chin rest. At the beginning of the experiment, the eye to be tracked was determined from the quality of calibration. A 9-point calibration was done for calibration points at the center, four corners and mid-points of the edges of the stimulus display area. A tolerance of 2° was maintained for error between calibration and validation. If the error was larger, calibration was repeated. For participants with consistently poor calibration or validation in the left eye, the right eye was tracked. For 14 of the 19 participants whose data were finally analyzed, the left eye was tracked.

Before each trial, drift correction was performed during the fixation display automatically by the EyeLink system. If drift was greater than 2°, the trial did not proceed and calibration was repeated. Calibration was also done at the start of each block.

During the experiment, EEG was also recorded, but these data will be reported elsewhere.

### Analysis of data

#### Eye movements

Fixation locations, fixation durations, and pupil sizes were determined and output by the EyeLink software. This software detected saccades based on an eye velocity threshold of 22°/s and an acceleration of 3800°/s^2^.

Since calibration was performed only once before a block, which lasted about 9 min, and the head was fixed with a chin rest only, errors in eye location because of head movements cannot be excluded. To check location accuracy, we visually inspected scan paths for each participant both trial-by-trial and by superimposing scan paths of all trials in each block over the rectangular viewing area. To make corrections in fixation locations, all targets from all trials in a block were centered and superimposed. For fixations within 3.12° around the target, a fixation density array was generated and a Gaussian filter was applied to generate a heat map. A heat map that was not centered at the target indicated a shift in the recorded eye location. The vector from the center of mass of the heat map to the center of the target was used as a correction factor for all fixation locations in that block. 2 of 23 participants who had excessive eye movements outside the display in spite of the corrections were excluded from analysis.

During analysis of fixations, a target was considered detected when a fixation was located within 2° radius around the target. Körner and Gilchrist (2007) showed that for stimuli with bounding circles that reduced discriminability similar to the ones used in our experiment, fixation distances beyond 3° reduced item detection to chance levels. Moreover, since no difference had previously been found between a 2° criterion and a criterion computed with a nearest neighbor algorithm for assigning fixations to visual search targets (McCarley et al., 2006), we used a criterion of 2°.

#### Fixation duration and pupil size

Fixation duration and pupil size recorded during the *search display* were analyzed for trials with correct responses. We divided the 10 s duration of search interval into bins of 1 s each. For each participant, in each bin, fixation durations were averaged across fixations on both targets and distractors.

Pupil size was analyzed during fixations and additionally as a time series. In fixations, the sampling points of pupil size were first averaged in time within each fixation and then, across all fixations in the bin. Before both analyses, the pupil signal was preprocessed. Since pupil response is known to be slow while the eye tracker is a source of high-frequency noise (Klingner et al., 2008), the pupil size signal was low-pass filtered at 10 Hz to remove noise from the eye tracker. For blinks detected by the EyeLink software, 10 samples before the blink, all samples during the blink, and 20 samples after the blink were removed and replaced by spline interpolation. Fewer than 1% of samples were replaced in this way. Other spike-like artefacts probably related to partial occlusion of the pupil by eyelids were detected by custom-made software and also replaced by spline interpolation. On average, 3.26% of the data was replaced in this process. For each participant, pupil sizes series were normalized by finding the mean pupil size during the search display irrespective of conditions and subtracting this mean value from each sample point.

#### Statistical analysis

We used repeated-measures ANOVA with the Huynh-Feldt correction (ε) of *p*-values associated with two or more degrees of freedom in order to compensate for violation of sphericity. We used the Fisher’s LSD (least significant difference) test for *post-hoc* analyses.

## Results

### Performance

The average accuracy in the task was greater than 70% (Table 1).

**Table 1:** **The percentage of correct trials for 19 participants**.

Targets	*Different* trials		*Same* trials		All trials	
	M	SD	M	SD	M	SD
3	80.8	8.47	87.3	6.92	84.1	7.19
4	72.23	10.06	81.6	10.95	76.9	9.54
5	68.5	11.16	79.1	11.17	73.8	9.43
All	73.9	8.65	82.7	8.77	78.3	8.1

The accuracy in *same* trials was higher than that in *different* trials (*F*_(1,18)_ = 36.4, *p* < 0.001). Accuracy decreased with the number of targets, as indicated by the effect of target (*F*_(2,36)_ = 30.3, *p* < 0.001, ε = 0.96). Accuracy was higher for 3-target condition compared to 4-target condition (*p* < 0.001) and 5-target condition (*p* < 0.001). There was no difference in accuracy between 4- and 5-target conditions.

A correct response does not mean that all the targets presented were fixated. Participant could score well above chance level by inspecting a few targets and gauge the rest from peripheral vision, or even guess the rest. Since we could not eliminate either of these possibilities, we grouped trials according to the number of targets fixated irrespective of the number presented. This way, a target condition corresponded to the number of targets that were fixated and most likely attended and memorized.

When initially using the criterion that a target fixation should last longer than 200 ms, some participants had very few trials in the 5-target condition. Therefore, we took into account the minimum number of trials needed in each condition to determine the criterion for target fixation duration. Histograms of fixation durations within 2° of the target peaked between 120 ms and 180 ms. In a range of 10–20 for number of trials and 120 ms–150 ms for fixation duration, we generated exhaustive combinations of number of trials and fixation duration. We used each combination of number of trials and target fixation duration as a criterion to find the number of participants satisfying this criterion. We found that for a criterion of 10 for number of trials and 150 ms for target fixation duration, the maximum number of participants satisfied the criterion. Data from two participants who could not meet this criterion were excluded from analysis, leaving us with data for the 19 participants that we finally analyzed.

Since we presented a minimum of 3 targets, we analyzed only those trials where at least 3 targets were fixated. In the 3-target condition all the targets presented were always fixated. For the 4- and 5-target condition, in 94.2% and 89.6% of *different* trials, the changed target was fixated and in 58.3% and 36.2% of the *same* trials, all presented targets were fixated, respectively. This result confirms that even when participants responded correctly, they did not always fixate on the necessary targets.

**Figure 2:**
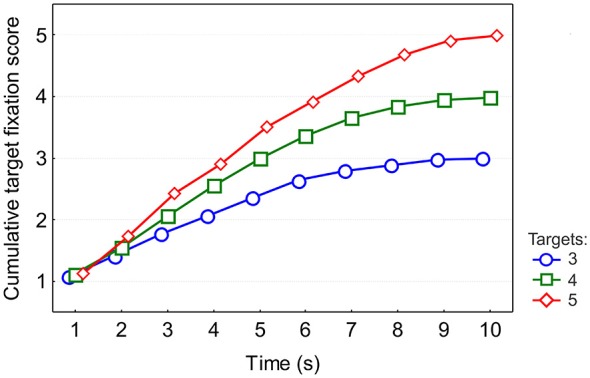
**Cumulative target fixation scores calculated for each second of search interval**. The means across 19 participants are shown. Standard errors are not displayed because they were so small that they did not exceed the mean markers in size.

To evaluate the time course of target fixations in a trial, we calculated the average cumulative target fixation scores as the number of targets fixated up to each second of the search interval (Figure 2). An ANOVA with target condition and time as factors showed a significant effect of target (*F*_(2,36)_ = 1605.9, *p* < 0.001, ε = 0.58), an effect of time (*F*_(9,162)_ = 971.4, *p* < 0.001, ε = 0.28) and an interaction between target and time (*F*_(18,324)_ = 128.6, *p* < 0.001, ε = 0.6).

### Fixation duration vs. pupil size

**Figure 3:**
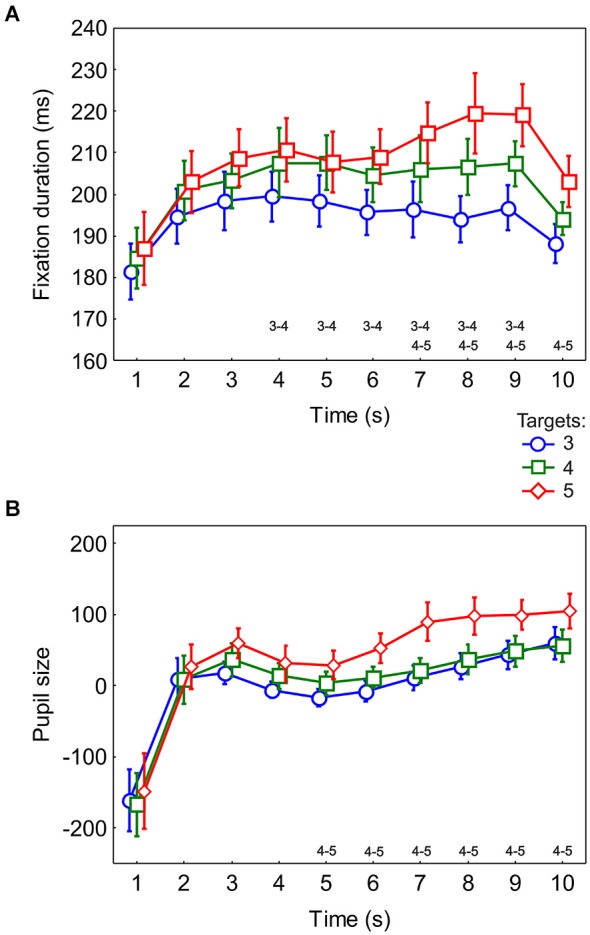
**Average fixation duration and pupil size**. Fixation duration **(A)** and pupil size (arbitrary units) **(B)** in 1-s intervals for the entire search interval. Along the time axis the target conditions having significant difference are indicated as found from *post-hoc* tests. The difference between 3- and 5-target conditions is not shown because it occurred from the 2nd to the 10th second for fixation duration and from the 3rd to the 10th second for pupil size. All plots show the data averaged across 19 participants. The error bars indicate the standard errors of the means.

We applied an ANOVA with factors of time and target condition on fixation duration and pupil size. There was an effect of time for both fixation duration (*F*_(9,162)_ = 13.99, *p* < 0.0001, ε = 0.4) (Figure 3A) and pupil size (*F*_(9,162)_ = 7.9, *p* < 0.001, ε = 0.24) (Figure 3B). There was a prominent effect of target condition for fixation duration (*F*_(2,36)_ = 50.4, *p* < 0.0001, ε = 0.71) and *post-hoc* tests showed a significant increase in fixation duration with number of targets (all *p* < 0.001). For pupil size, the effect of target condition was significant (*F*_(2,36)_ = 4.3, *p* = 0.02, ε = 0.95) with *post-hoc* tests showing that pupil size was larger for the 5-target condition than the 3-target condition (*p* < 0.01) and the 4-target condition (*p* < 0.05).

There was an interaction between target condition and time for fixation duration (*F*_(18,324)_ = 2.3, *p* = 0.04, ε = 0.31). *Post-hoc* tests showed the emergence of a difference between 3- and 5-target conditions beginning at two seconds and staying until the end of search, a difference between 3- and 4-target conditions emerging at the 4th second and staying till the 9th second and a difference between 4- and 5-target conditions beginning at the 7th second and staying till the end of search (Figure 3A). For pupil size the interaction between target condition and time only approached significance (*F*_(18,324)_ = 2, *p* = 0.06, ε = 0.36) though it was significant (*p* = 0.009) before correcting for sphericity. *Post-hoc* tests revealed a difference between 3- and 5-target conditions beginning from the 3rd second and staying until the end of search, no difference between 3- and 4-target conditions and a difference between 4- and 5-target conditions from the 5th second till the end of search (Figure 3B).

Thus, both fixation duration and pupil size showed sensitivity to the number of targets in the display. In contrast to pupil size, fixation duration showed a difference between the target conditions within memory capacity (3 and 4). This difference lasted for 6 s of the entire interval of visual search, suggesting sensitivity of fixation duration to memory load. In the middle of the interval, after about 5–6 s, a difference between target conditions above memory capacity (4 and 5) appeared. This effect occurred simultaneously in both measures, suggesting a late onset of additional processing load effects.

In addition to the assessment of pupil size within fixations we analyzed the evolution of pupil size over all 2500 sampling points. We applied an ANOVA with 3 targets as a factor on each of the 2500 sampling points. The false discovery rate was controlled using the procedure described by Storey (2002).

**Figure 4:**
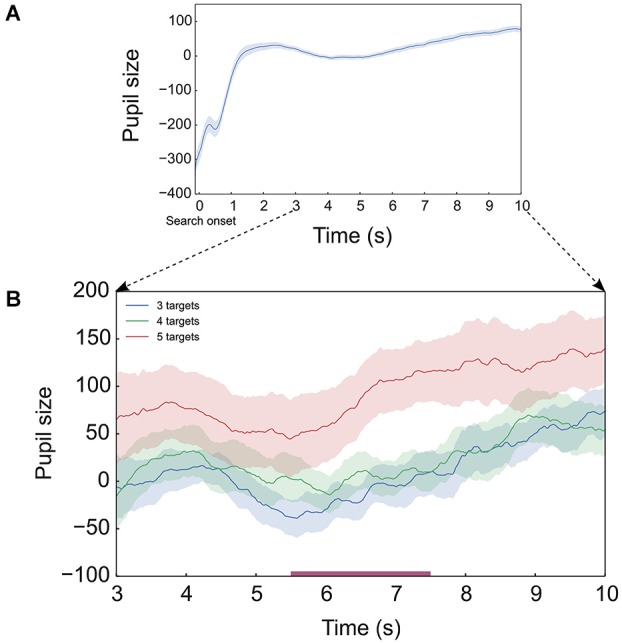
**Pupil size series (arbitrary units). (A)** Pupil size series averaged across all target conditions for entire duration of search interval. **(B)** Pupil size series for the 3 target conditions after omitting the initial pupil response to luminance change. In **(B)** the bar along the time axis indicates the interval of significant difference between target conditions as found in the point-wise ANOVA. All plots show the pupil size series averaged across 19 participants. The shaded areas indicate standard errors of the means.

In the pupil size series, there was a steep increase in the first 1.5 s, (Figure 4A), which could be attributed to luminance change from the fixation cross to the onset of the *search display*. After this time, pupil size generally increased throughout the search interval. Figure 4B illustrates this increase after omitting the initial pupil response to luminance change. The point-wise ANOVA showed a main effect of target in the interval 5.5–7.5 s, which corresponds to the maximal deviation of the 5-target condition. This result indicates that an effect of target on pupil size occurs, albeit only for a limited time interval.

### Targets vs. distractors

To evaluate the contribution of targets and distractors to the effect of target condition, we assessed fixation duration and pupil size separately for target and distractor fixations. We pooled the 3-, 4-, and 5-target conditions and distinguished target fixations based on order of visiting during search into 1st, 2nd, 3rd and 4th target visited. Targets visited 5th were not considered because their number was too small. To have the same amount of target and distractor data, for each target we selected the fixation on either the preceding or the following distractor alternatingly, after having established that these two do not differ systematically. Fixation duration and pupil size were averaged for each target visiting order, separately for target and distractor fixations. Only first target fixations were used for this analysis. It could not be excluded, however, that some of the preceding and following distractor fixations are re-fixations.

**Figure 5:**
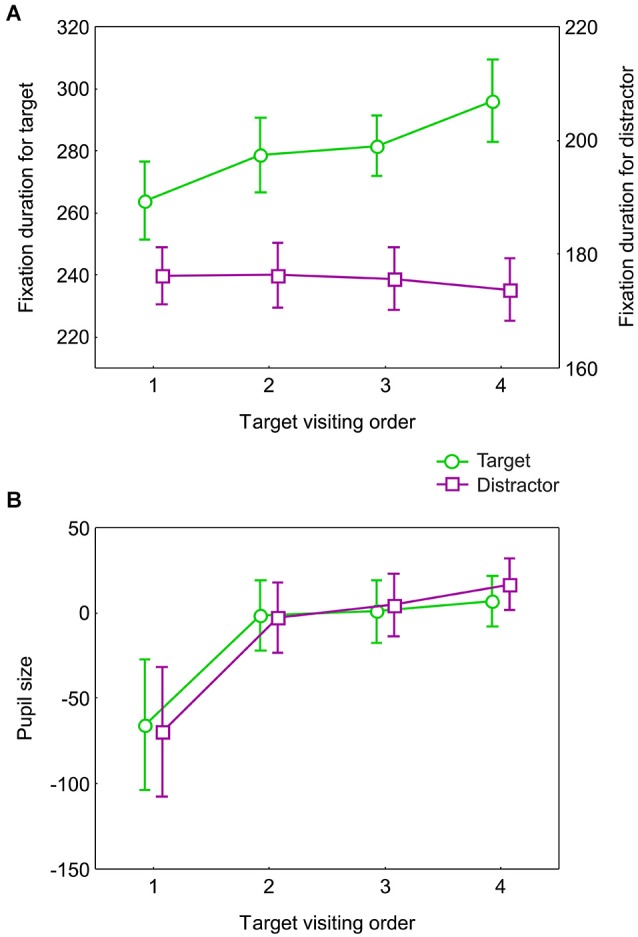
**Fixation duration and pupil size for targets and distractors**. Fixation duration **(A)** and pupil size (arbitrary units) **(B)** for fixations on successive targets and their preceding distractors in the order of target fixations. Only for fixation duration, a significant difference between targets and distractors as found from *post-hoc* tests was seen for all target visiting orders. All plots show the data averaged across 19 participants. The error bars indicate standard errors of the means.

We performed an ANOVA with fixation type (target vs. distractor) and visiting order (1st, 2nd, 3rd, and 4th) as factors. Target fixations were much longer than distractor fixations (*F*_(1,18)_ = 149.1, *p* < 0.0001) (Figure 5A). But there was no fixation type effect on pupil size. There was an effect of target visiting order for fixation duration (*F*_(3,54)_ = 4.1, *p* = 0.01, ε = 1), while for pupil size this effect did not reach significance (*F*_(3,54)_ = 2.3, *p* = 0.08) despite visible increase of pupil size between 1st and 2nd targets (Figure 5B). From the cumulative target scores we know that the first target is fixated by the first second (Figure 2), in which time, pupil size responds predominantly to luminance changes (Figure 4A). This suggests that the pupil size during the fixation on the 1st target is defined by luminance and it is unlikely that it reflects any difference between targets and distractors related to cognitive processing.

For fixation duration there was a significant interaction between fixation type and visiting order (*F*_(3,54)_ = 9.6, *p* < 0.0001, ε = 1). *Post-hoc* tests showed that for targets there was a significant increase in fixation duration with target visiting order between all target visiting order combinations except between 2nd and 3rd targets (all *p* < 0.01). For distractors, there was no difference between target visiting orders. *Post-hoc* tests also revealed highly significant differences between targets and distractors for all target visiting orders (all *p* < 0.001) (Figure 5A). We tested the changes across the target visiting orders using a planned comparison with linear contrast over visiting order. There was a linear trend for targets (*F*_(1,18)_ = 18.6, *p* < 0.001) but not for distractors (*p* = 0.2). In sum, fixation duration increases with visiting order only for targets and not for distractors.

Pupil size showed no interaction between fixation type and target visiting order. Pupil size, therefore, reveals no difference between targets and distractors. Pupil size, being a slow signal, is hence ineffective in differentiating eye fixations on targets and distractors in free viewing.

## Discussion

We asked how memory load affects fixation durations and pupil size in free viewing behavior. To this end, we recorded eye movements and pupil size in a multiple-targets *visual search* task in which participants could freely move their eyes. The accuracy of memory for targets was tested in a subsequent *change detection* task where participants detected the change in orientation of one of the targets. The effect of memory load on fixation duration and pupil size was evaluated in the course of the *visual search* task. The number of targets (3, 4 and 5) fixated in the *search display* was an index of memory load. Fixation durations and pupil size were also compared for successive target and distractor visits.

Our findings suggest that the 10 s of the visual search task can be approximately divided in two sequential stages of processing: the first stage is dominated by loading targets into memory and in the second stage memory accumulation is accompanied by other cognitive processes. The following line of evidence supports this conclusion.

The first 4 targets used in the targets vs. distractors analysis were visited within 6 s (Figure 2). In this interval there was a linear increase in the duration of fixation on targets but not on distractors (Figure 5A). Later during this interval (4–6 s), fixation duration was longer for 4- than 3-target conditions, both within the memory capacity limit. In the following interval, about 7–10 s, both fixation duration and pupil size increase with time and both differentiate between 4 and 5 target conditions, i.e., above the memory capacity limit (Figure 3). Thus, the initial difference in fixation duration within the memory capacity was associated with increasing duration of target fixations, whereas the later part with the number of targets above memory capacity was additionally characterized by still longer fixation duration and larger pupil size.

### Fixation duration

Cumulative target fixation scores (Figure 2) show that targets are fixated sooner in conditions which have more targets, leading to a prominent difference between the three conditions at any time. This pattern is discernible in the dynamics of fixation duration. The deviation in fixation duration between 3- and 5-target conditions starts at 2 s, followed by a deviation between 3- and 4-target conditions. The early increase in fixation duration coincides with the early increase in the number of targets fixated (Figure 2). As well, the different rates of increase of fixation duration for 3-, 4- and 5- target conditions (Figure 3A) mirror the rates of increase in the number of targets fixated (Figure 2). The difference between 3- and 4-target conditions, in which the numbers of targets are still within the limit of working memory capacity, indicates sensitivity of fixation duration to memory load. The fixation durations in the 4- and 5-target conditions begin to differ only from the 7th second onwards (Figure 3A) when in the 5-target condition 4 targets have already been seen. Assuming memory is fully loaded at this time, the following prominent increase in the 5-target condition may reflect cognitive control operations involved in managing the number of items exceeding the capacity limit.

We observed a huge difference in fixation duration between targets and distractors. This is not surprising, because after detection of targets attention stays on targets for encoding, while after detection of distractors the focus of attention shifts away.

The target-distractor comparison revealed that the observed difference between the 3-, 4-, and 5-target conditions occurs because of the increasing fixation durations on successive targets but not on distractors (Figure 5A). No increase in duration for distractor fixations indicates that the effect is specific to accumulating targets into memory rather than decreasing resources or increasing cognitive effort while performing the task.

A general trend of fixation durations increasing with time is known to occur because of transition from global to local scanning strategies and is typically limited to the first 2 s of free visual exploration (Unema et al., 2005). Such an effect was seen for all of 3-, 4- and 5-target conditions (Figure 3A). The effects suggesting memory accumulation are reflected later both in the difference between 3-, 4- and 5-target conditions and in the linear trend for successively visited targets.

The increase of fixation duration with memory load is consistent with the results of previous studies of multiple target visual search (McCarley et al., 2006), single target visual search (Peterson et al., 2008; He and McCarley, 2010) and comparative search tasks (Inamdar and Pomplun, 2003).

Fixation duration, thus, reflects memory load precisely when the number of items lies within memory capacity limit. In the second half of the search interval in our task, when memory load exceeds the memory capacity, fixation duration probably indicates the effect of processing load in addition to memory load. These results show no support for Model I (Bowman and Wyble, 2007; Wyble et al., 2011), however, they are in accordance with Model II (Simione et al., 2012; Raffone et al., 2014). As predicted by Model II, fixation duration divulges the dichotomy between memory load and processing load in the course of the search interval. An early difference occurs between 3- and 4-target conditions and between 3- and 5-target conditions indicating increase in memory load within the memory capacity. This corresponds to the filling of the temporary buffer in Model II. According to Model I, a difference should have been seen between 4- and 5-target conditions as well at this stage, but no evidence bearing this was found in our results. Model II also posits that information that is filtered by attention occupies the temporary buffer for later consolidation into working memory. Such filling of the temporary buffer is supported by the increase in duration of fixations on successive targets and not on distractors. As memory load exceeds capacity limit, the difference between 4 and 5-target conditions emerges, when, according to Model II, processing load is heightened because encoding becomes more effortful.

### Pupil size

For 7 s of the entire search duration, pupil size showed a difference between 3- and 5-target conditions. 4- and 5-target conditions deviated from the 5th second onwards (Figure 3B). This coincides with the onset of the difference between 4- and 5-target conditions in fixation duration (Figure 3A). The cumulative target fixation score (Figure 2) indicates that at this moment the 5-target condition exceeded memory capacity of 4. The pupil size series also showed an effect of number of targets only in this interval of maximal deviation of the 5-target condition.

Pupil size does not differ between targets and distractors (Figure 5B). Furthermore, pupil size does not change with target visiting order. Its increase during the search interval is most prominent after 5 s (Figure 3B). These findings constitute additional evidence that pupil size is unlikely to be related to memory load and probably reflects cognitive effort which is most prominent in the second half of our task.

Porter et al. (2007) also reported an increase of pupil size with time in free viewing. In this study, the pupil dilated quickly and sustained till the end of a counting task, while it dilated gradually throughout a visual search task. These different dilatory patterns were ascribed to the differences in spatial memory requirements between the tasks. In our experiment, a similar gradual increase in pupil size in time is seen (Figures 3B, 4), which might correspond to spatial memory requirements imposed by the visual search task for tagging of found targets and visited distractors (Shore and Klein, 2000; Körner and Gilchrist, 2008). Besides this, when memory load increased within the task, pupil size did not appear to have the temporal resolution necessary to observe memory load changes at the level of a single fixation.

Thus, pupil size reflects the number of targets only when it exceeds the memory capacity limit. This suggests that pupil size reflects processing load rather than memory load. In our task, the processing load might involve multiple collateral processes accompanying memory management. This additional effort required for encoding in the case of 5 targets, which is reflected in pupil size, was predicted by Model II as the late emergence of difference between 4 and 5 targets.

## Conclusion

In sum, our findings indicate that both fixation duration and pupil size depend on the number of targets which are fixated in a search task with unrestricted eye movements. Fixation duration is selective to memory load for targets. In contrast, pupil size is too slow for isolating instances of memory accumulation such as target encoding in the free viewing search task. Pupil size most likely reflects an overall processing load which incorporates several cognitive processes. The slowness of pupil size dynamics renders it unlikely that a combination of fixation duration and pupil size may enhance our understanding of memory accumulation, compared to fixation duration alone.

The dynamics of the search task and the different target-distractor processing are in good correspondence with the predictions of Model II. This could be understood as evidence for involvement of a temporary buffer in memory accumulation of multiple targets across sequential fixations in free viewing behavior.

## Conflict of interest statement

The authors declare that the research was conducted in the absence of any commercial or financial relationships that could be construed as a potential conflict of interest.
